# Platelet Dysfunction in Patients with Chronic Myeloid Leukemia: Does Imatinib Mesylate Improve It?

**DOI:** 10.4274/tjh.2014.0213

**Published:** 2016-05-16

**Authors:** Olga Meltem Akay, Fezan Mutlu, Zafer Gülbaş

**Affiliations:** 1 Osmangazi University Faculty of Medicine, Department of Hematology, Eskişehir, Turkey

**Keywords:** Platelet aggregation, Chronic myeloid leukemia, Imatinib mesylate

## Abstract

**Objective:**

The aim of this study was to investigate the effects of imatinib mesylate on platelet aggregation and adenosine triphosphate (ATP) release in chronic myeloid leukemia patients.

**Materials and Methods:**

Platelet aggregation and ATP release induced by 5.0 mM adenosine diphosphate, 0.5 mM arachidonic acid, 1.0 mg/mL ristocetin, and 2 µg/mL collagen were studied by whole blood platelet lumi-aggregometer in 20 newly diagnosed chronic myeloid leukemia patients before and after imatinib mesylate treatment.

**Results:**

At the time of diagnosis, 17/20 patients had abnormal platelet aggregation results; 8 (40%) had hypoactivity, 6 (30%) had hyperactivity, and 3 (15%) had mixed hypo- and hyperactivity. Repeat platelet aggregation studies were performed after a mean of 19 months (min: 5 months-max: 35 months) in all patients who received imatinib mesylate during this period. After therapy, 18/20 (90%) patients had abnormal laboratory results; 12 (60%) had hypoactive platelets, 4 (20%) had mixed hypo- and hyperactive platelets, and 2 (10%) had hyperactive platelets. Three of the 8 patients with initial hypoactivity remained hypoactive, while 2 developed a mixed picture, 2 became hyperactive, and 1 normalized. Of the 6 patients with initial hyperactivity, 4 became hypoactive and 2 developed a mixed pattern. All of the 3 patients with initial hypo- and hyperactivity became hypoactive. Finally, 2 of the 3 patients with initial normal platelets became hypoactive while 1 remained normal. There was a significant decrease in ristocetin-induced platelet aggregation after therapy (p<0.001), while platelet aggregation and secretion induced by other agonists showed no difference after treatment (p>0.05).

**Conclusion:**

These findings indicate that a significant proportion of chronic myeloid leukemia patients have different patterns of platelet function abnormalities and imatinib mesylate has no effect on these abnormalities, with a significant impairment in ristocetin-induced platelet aggregation.

## Abstract (tr)

**Amaç:**

Bu çalışmanın amacı kronik miyelositer lösemili hastalarda imatinib mesilatın trombosit agregasyonu ve adenozin trifosfat (ATP) salınımı üzerine etkilerini araştırmaktır.

**Gereç ve Yöntemler:**

Yirmi yeni tanı almış kronik miyelositer lösemili hastada imatinib mesilat tedavisi öncesi ve sonrası 5,0 mM adenozin difosfat, 0,5 mM araşidonik asit, 1,0 mg/mL ristosetin ve 2 µg/mL kollagen ile indüklenen trombosit agregasyon ve ATP salınımı çalışılmıştır.

**Bulgular:**

Tanı sırasında, 17/20 hasta anormal trombosit agregasyon sonuçlarına sahip idi; sekizinde (%40) hipoaktivite, altısında (%30) hiperaktivite ve üçünde (%15) miks hipo- ve hiperaktivite saptandı. Ortalama 19 ay (min: 5 ay-maks: 35 ay) imatinib mesilat kullanımı sonrası tüm hastalarda trombosit agregasyon testleri tekrarlandı. Tedavi sonrası, 18/20 (%90) hasta anormal laboratuvar sonuçlarına sahip idi; 12’si (%60) hipoaktif trombositler, dördü (%20) miks hipo- ve hiperaktif trombositler ve ikisi (%10) hiperaktif trombositlere sahip idi. Başlangıçta hipoaktivitesi olan sekiz hastanın üçü hipoaktif kalır iken ikisi miks bir görüntü geliştirdi, ikisi hiperaktif oldu ve biri normalize oldu. Başlangıçta hiperaktivitesi olan altı hastanın dördü hipoaktif oldu ve ikisi miks patern geliştirdi. Başlangıçta hipo- ve hiperaktivitesi olan üç hastanın tamamı hipoaktif oldu. Son olarak, başlangıçta normal trombositleri olan üç hastanın ikisi hipoaktif olur iken biri normal kaldı. Ristosetin ile indüklenen trombosit agregasyonunda tedavi sonrası anlamlı azalma (p<0,001) olur iken diğer agonistler ile indüklenen trombosit agregasyon ve sekresyonu tedavi sonrası farklılık göstermedi (p>0,05).

**Sonuç:**

Bulgularımız kronik miyelositer lösemili hastaların önemli bir çoğunluğunun farklı paternde trombosit fonksiyon anormalliklerine sahip olduğunu ve imatinib mesilatın ristosetin ile indüklenen trombosit fonksiyonunda azalma dışında bu anormallikler üzerinde etkisi olmadığını göstermiştir.

## INTRODUCTION

Imatinib mesylate (Gleevec or Glivec, Novartis, Basel, Switzerland) is the first tyrosine kinase (TK) inhibitor active against BCR-ABL, c-ABL, ARG, PDGF-r, and c-KIT. TKs are important signaling enzymes for the cellular regulation of proliferation, differentiation, survival, function, and motility, and various tumors overexpress TKs, leading to uncontrolled mitogenic signals to neoplastic cells [1]. Imatinib mesylate has considerable antineoplastic activity in patients with chronic myeloid leukemia (CML) and some solid tumors [2].

Thromboembolic and bleeding complications are the leading causes of morbidity and mortality in myeloproliferative neoplasms, particularly polycythemia vera and essential thrombocythemia, although these occur least frequently in patients with CML [3]. Abnormalities of platelet function arising from the clonal proliferation of hematopoietic cells including megakaryocyte precursors are regarded as the main origin of thrombo-hemorrhagic episodes [4]. Considering the reduction of BCR-ABL+ clones in response to imatinib mesylate and the recovery of normal hematopoietic stem and progenitor cells in the bone marrow [5], we performed platelet aggregation studies in CML patients who were treated with imatinib mesylate to investigate the effect of this drug on platelet function.

## MATERIALS AND METHODS

### Patients

A total of 20 newly diagnosed chronic-phase CML patients who started treatment with imatinib were enrolled. The diagnosis of CML was made by the demonstration of Philadelphia chromosome positivity and compatible hematological findings in peripheral blood and bone marrow. Imatinib was used as the fırst-line treatment in all patients. It was started at a dose of 400 mg daily. Dose modifications were allowed according to toxicity and treatment efficacy, ranging from 200 to 800 mg.

### Sample Collection

Venous blood was collected from patients under light tourniquet through 19-gauge needles into vacutainers (Becton Dickinson). A 3-mL di-potassium EDTA (1.5 mg/mL) sample was collected fırst followed by two 4.5-mL 3.2% tri-sodium citrate (0.105 M) vacutainers. The collection was performed early in the morning following a light breakfast. Subjects with known bleeding or other systemic disorders such as renal, hepatic, and endocrine diseases, and those who had taken aspirin or other nonsteroidal anti-inflammatory agents within 10 days prior to blood sampling were excluded. Automated cell counts were performed on the EDTA sample tube with a Beckman Coulter Gen-S SM (USA) automated blood-counting device.

### Whole Blood Platelet Lumi-Aggregometry

Whole blood platelet lumi-aggregometry studies were performed on the citrate tubes at the time of diagnosis and repeated following imatinib therapy in all patients. Platelet aggregation (measured as the increase in impedance) and release were simultaneously measured using a whole blood lumi-aggregometer (Model 560-Ca, Chrono-log Corporation, USA) according to the manufacturer’s instructions. The agonists used and their final concentrations were, in sequence, adenosine diphosphate (ADP; Chrono Par 384, Chrono-log Corporation), 5 µM; arachidonic acid (AA; Chrono Par 390), 0.5 mM; ristocetin (Chrono Par 396), 1.0 mg/mL; and collagen (Chrono Par 385), 2 µg/mL. Platelet function testing on all samples was completed within 2 h of collection. Our laboratory reference ranges for platelet aggregation (ohm) and adenosine triphosphate (ATP) release (nmol) were 10-22 ohm and 0.3-2 nmol for ADP, 10-28 ohm and 0.6-3 nmol for AA, 10-32 ohm and 0.3-2 nmol for collagen, and 3-19 ohm for ristocetin.

### Statistics

Statistical analysis was performed using IBM SPSS 20.0 (IBM Corp., Armonk, NY, USA). The Shapiro-Wilk test was used to test normality for continuous variables. Nonnormally distributed variables were compared with the Wilcoxon test for paired data and presented as medians (quartiles). P<0.05 was accepted as statistically significant.

## RESULTS

The median age of the total group of patients was 43 years (range: 29-71); there were 10 females and 10 males. Median platelet counts before and after treatment were 339.50x109/L (range: 227.50-527.25) and 225.50x109/L (range: 171.00-259.25), respectively.

As described by Manoharan et al. [6], platelets were considered to be hyperactive if at least one result (aggregation or ATP release with one agonist) was above the reference range and hyporeactive if at least one result (aggregation or ATP release with one agonist) was below the reference range. Mixed hypo- and hyperactive platelets were considered present when at least one result (aggregation or ATP release) was below and above the reference range, respectively.

At the time of diagnosis, 17/20 patients had abnormal platelet aggregation results; 8 (40%) had platelet hypoactivity, 6 (30%) had platelet hyperactivity, and 3 (15%) had mixed hypo- and hyperactivity.

**Figure 1:**
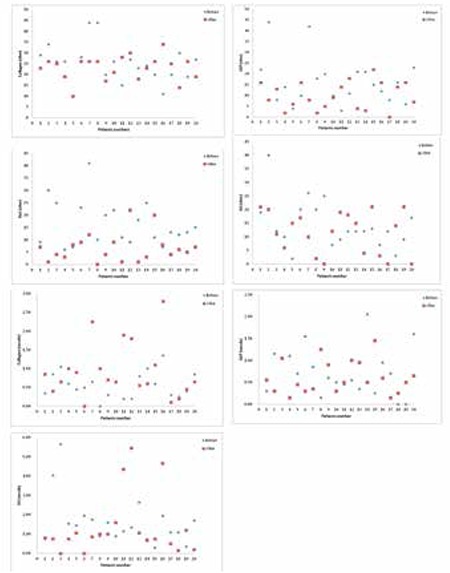
Platelet aggregation (ohm) and secretion (nmol) values induced by agonists before and after imatinib therapy in CML patients (n=20). Reference interval for impedance (ohm) or release (nmol) of the agonist is shown by dotted lines in each chart. ADP: Adenosine diphosphate, AA: arachidonic acid.

After a mean of 19 months (min: 5 months-max: 35 months), repeat platelet aggregation studies were performed in all patients who received imatinib during this period. A major molecular response was achieved in 17 (85%) of the patients at the time of retesting. After imatinib therapy, 18/20 (90%) patients had abnormal laboratory results; 13 (65%) had hypoactive platelets, 3 (15%) had mixed hypoa and hyperactive platelets, and 2 (10%) had hyperactive platelets. Three of the 8 patients with initial hypoactivity remained hypoactive, while 2 developed a mixed picture, 2 became hyperactive, and 1 normalized. Of the 6 patients with initial hyperactivity, 5 became hypoactive and 1 developed a mixed pattern. All of the 3 patients with initial hypo- and hyperactivity became hypoactive. Finally, 2 of the 3 patients with initial normal platelets became hypoactive while 1 remained normal (Figure 1).

When we compared pretreatment and post treatment platelet aggregation values induced by ADP, AA, ristocetin, and collagen, we found that there was a significant decrease in ristocetin-induced platelet aggregation (p<0.001) after treatment, while pre- and post treatment platelet aggregation responses to the other agonists were not significantly different (p>0.05). There was also no significant difference between pretreatment and post treatment platelet secretion values induced by ADP, AA, and collagen (Table 1). We did not notice a significant correlation between platelet count and platelet aggregation and secretion results induced by any of the agonists used. Moreover, platelet responses showed no correlation with the length of time on imatinib and none of the studied patients experienced bleeding.

## DISCUSSION

In the present study, we demonstrated that a significant proportion of CML patients (85%) have different patterns of platelet dysfunction and imatinib therapy has neither a positive nor a negative impact on these functional defects.

The number of studies investigating the effect of CML therapy on platelet abnormalities is very limited. In one study including 6 CML patients, plasma levels of beta-TG and PF4 were not reduced and platelet aggregation did not improve following normalization of leukocyte and platelet counts after busulfan or hydroxyurea [7]. In contrast, Barbui et al. [8] reported a normalization of spontaneous platelet aggregation and improvement of collagen-induced aggregation but a persistent dense granule storage deficiency after busulfan therapy. Data evaluating the effects of imatinib on platelet function are also still limited. In a recent study by Quintas-Cardama et al. [9], 5 of the 15 evaluable CML patients on imatinib had normal platelet aggregation and 10 (66%) had impaired AA-induced platelet aggregation, including 2 (13%) with impaired epinephrine-induced aggregation.

Our results suggest that imatinib does not have either a positive or a negative impact on platelet function, with a significant impairment in ristocetin-induced platelet aggregation. It is known that, during ristocetin-induced platelet aggregation, ristocetin binds to the platelet surface through its phenolic groups. Being positively charged, the bound ristocetin reduces the net negative charge on the platelet surface and permits a closer contact between platelets. This, in turn, permits the von Willebrand factor to bridge between platelets, resulting in agglutination. Imatinib, either by altering ristocetin’s phenolic groups or by occupying its binding sites on the platelet surface, may cause a decrease in ristocetin-induced platelet aggregation. Moreover, since ristocetin does bind to the platelet, the decrease in platelet counts after imatinib treatment may be another explanation for impaired ristocetin-induced platelet aggregation results. However, these hypotheses must be confirmed with further in vitro studies [10,11].

We speculated that myelosuppression under imatinib treatment depends on the fact that after reduction of clonal hematopoiesis in response to treatment, normal hematopoietic stem and progenitor cells have to recover from preexisting suppression by the malignant clone and re-expand in the bone marrow. This may be related to changes in the growth pattern of megakaryocytes and a certain improvement of platelet function and activation after therapy. However, imatinib treatment did not improve most of the patterns of platelet function abnormalities while significantly increasing hypoactivity of platelets. The existence of platelet function abnormalities even in patients who achieved a major molecular response with imatinib in our study led us to assume that normal hematopoiesis is not fully restored in a substantial portion of CML patients despite the achievement of the desired response. Imatinib mesylate is designed as a TK inhibitor active against BCR-ABL [12,13], but it also inhibits other TKs, such as PDGF-r and c-KIT [14,15]. Inhibitory effects of the drug on platelet function may be partly explained by the inhibition of platelet TKs.

## CONCLUSION

In conclusion, these findings indicate that a significant proportion of CML patients have different patterns of platelet function abnormalities, which must be further investigated. However, one important limitation of this study is that we only studied platelet function tests in CML patients without a control group including imatinib-treated non-CML patients such as patients with gastrointestinal stromal tumors or hypereosinophilic syndrome. Subsequent studies should investigate how imatinib changes the function of platelets in patients with normal hematopoiesis.

## Ethics

Ethics Committee Approval: Eskişehir Osmangazi University Ethics Committee, Informed Consent: It was taken.

## Figures and Tables

**Table 1:**
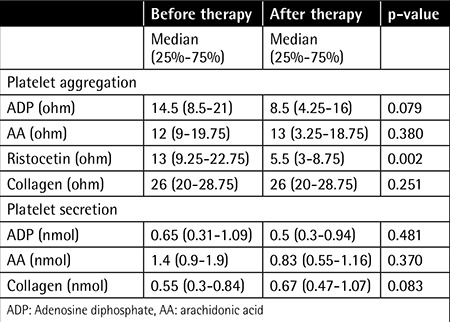
Comparison of platelet aggregation and secretion results induced by agonists before and after imatinib therapy in chronic myeloid leukemia patients (n=20).

## References

[1] Hensley ML, Ford JM (2003). Imatinib treatment: specific issues related to safety, fertility, and pregnancy. Semin Hematol.

[2] Agis H, Jaeger E, Doninger B, Sillaber C, Marosi C, Drach J, Schwarzinger I, Valent P, Oehler L (2006). In vivo effects of imatinib mesylate on human haematopoietic progenitor cells. Eur J Clin Invest.

[3] Schafer AI (1984). Bleeding and thrombosis in myeloproliferative disorders. Blood.

[4] Wehmeier A, Schneider W (1996). Megakaryocytes and platelets as the main cause for vascular events in chronic myeloproliferative disorders. Hamostaseologie.

[5] Appel S, Balabanov S, Brümmendorf TM, Brossart P (2005). Effects of imatinib on normal hematopoiesis and immune activation. Stem Cells.

[6] Manoharan A, Gemmell R, Brighton T, Dunkley S, Lopez K, Kyle P (1999). Thrombosis and bleeding in myeloproliferative disorders: identification of at-risk patients with whole blood platelet aggregation studies. Br J Haematol.

[7] Wehmeier A, Scharf RE, Fricke S, Schneider W (1990). A prospective study of hemostatic parameters in relation to the clinical course of myeloproliferative disorders. Eur J Haematol.

[8] Barbui T, Bassan R, Viero P, Cortelazzo S, Dini E (1983). Platelet function after busulfan in chronic myeloproliferative disorders. Haematologica.

[9] Quintas-Cardama A, Han X, Kantarjian H, Cortes J (;114:). Tyrosine kinase inhibitor-induced platelet dysfunction in patients with chronic myeloid leukemia. Blood 2009;114:.

[10] Coller BS, Gralnick HR (1977). Studies on the mechanism of ristocetin-induced platelet agglutination. Effects of structural modification of ristocetin and vancomycin. J Clin Invest.

[11] Kattlove HE, Gomez MH (1975). Studies on the mechanism of ristocetin-induced platelet aggregation. Blood.

[12] Druker BJ, Lydon NB (2000). Lessons learned from the development of an Abl tyrosine kinase inhibitor for chronic myelogenous leukemia. J Clin Invest.

[13] Druker BJ, Tamura S, Buchdunger E, Ohno S, Segal GM, Fanning S, Zimmermann J, Lydon NB (1996). Effects of a selective inhibitor of the Abl tyrosine kinase on the growth of BCR-ABL positive cells. Nat Med.

[14] Buchdunger E, Cioffi CL, Law N, Stover D, Ohno-Jones S, Druker BJ, Lydon NB (2000). Abl protein-tyrosine kinase inhibitor STI571 inhibits in vitro signal transduction mediated by c-kit and platelet-derived growth factor receptors. J Pharmacol Exp Ther.

[15] Heinrich MC, Griffith DJ, Druker BJ, Wait CL, Ott KA, Zigler AJ (2000). Inhibition of c-kit receptor tyrosine kinase activity by STI571, a selective tyrosine kinase inhibitor. Blood.

